# Combining streptozotocin and unilateral nephrectomy is an effective method for inducing experimental diabetic nephropathy in the ‘resistant’ C57Bl/6J mouse strain

**DOI:** 10.1038/s41598-018-23839-9

**Published:** 2018-04-03

**Authors:** Melissa Uil, Angelique M. L. Scantlebery, Loes M. Butter, Per W. B. Larsen, Onno J. de Boer, Jaklien C. Leemans, Sandrine Florquin, Joris J. T. H. Roelofs

**Affiliations:** Department of Pathology, Academic Medical Center, University of Amsterdam, Amsterdam, The Netherlands

## Abstract

Diabetic nephropathy (DN) is the leading cause of chronic kidney disease. Animal models are essential tools for designing new strategies to prevent DN. C57Bl/6 (B6) mice are widely used for transgenic mouse models, but are relatively resistant to DN. This study aims to identify the most effective method to induce DN in a type 1 (T1D) and a type 2 diabetes (T2D) model in B6 mice. For T1D-induced DN, mice were fed a control diet, and randomised to streptozotocin (STZ) alone, STZ+unilateral nephrectomy (UNx), or vehicle/sham. For T2D-induced DN, mice were fed a western (high fat) diet, and randomised to either STZ alone, STZ+UNx, UNx alone, or vehicle/sham. Mice subjected to a control diet with STZ +UNx developed albuminuria, glomerular lesions, thickening of the glomerular basement membrane, and tubular injury. Mice on control diet and STZ developed only mild renal lesions. Furthermore, kidneys from mice on a western diet were hardly affected by diabetes, UNx or the combination. We conclude that STZ combined with UNx is the most effective model to induce T1D-induced DN in B6 mice. In our hands, combining western diet and STZ treatment with or without UNx did not result in a T2D-induced DN model in B6 mice.

## Introduction

Diabetic nephropathy (DN) is the leading cause of chronic kidney disease^1^. DN is characterized by proteinuria, thickening of the glomerular basement membrane (GBM), glomerulosclerosis, and tubulo-interstitial fibrosis, which eventually result in end-stage renal disease. End-stage renal disease requires renal replacement therapies, such as transplantation and dialysis, which are associated with high healthcare costs and place huge burdens on patient’s quality of life. Current therapies to slow down DN progression primarily include metabolic control, blood pressure control and renin-angiotensin system (RAS) inhibition^2^. Although glycaemic control reduces the risk of DN, effective prevention of the development of DN remains an enormous challenge worldwide^3^. Animal models are essential tools for studying the molecular mechanisms of DN and are useful for the development of new therapeutic strategies to effectively prevent DN. The identification of suitable animal models of DN has become a priority of the US National Institutes of Health and led to the foundation of the Animal Models of Diabetic Complications Consortium (AMDCC) in 2001^4^. Researchers of the AMDCC characterized DN phenotypes in several mouse models. The streptozotocin (STZ)-induced type I diabetes model is by far the most commonly used model to induce DN, however, the onset of proteinuria and glomerulosclerosis appears to be strain dependent^5–9^. When considering non-genetic mouse models only, DBA/2(J) and KK/HIJ mice show the highest susceptibility to STZ-induced DN. These strains developed pronounced albuminuria, glomerulosclerosis, glomerular hypertrophy and GBM thickening. On the contrary, the most widely used C57Bl/6J (B6) strain was shown to be highly sensitive to the development of STZ-induced hyperglycaemia, however, its susceptibility to subsequent renal damage is low. Consequently, the B6 mouse strain is considered to be relatively resistant to the development of DN^4^.

Unilateral nephrectomy (UNx), in the context of diabetes, was first described by Steffes *et al*. STZ-induced diabetic Sprague-Dawley rats subjected to UNx showed enhanced renal injury compared to diabetic sham-operated rats^10^. A few years later, this model was applied to CD-1 mice as well^11,12^. In both studies, UNx accelerated the development of glomerular lesions and renal function decline in STZ-induced diabetic rodents, while UNx alone only induced low numbers of glomerular lesions and minimal renal function decline, suggesting a synergistic effect of STZ+UNx. Currently, comparative studies of the STZ and STZ+UNx models for type 1 diabetes (T1D)-induced nephropathy, specifically in B6 mice, are lacking.

B6 mice are also used as a model for type 2 diabetes (T2D), when combining a high fat diet (HFD) with STZ treatment^13^. When HFD and STZ are combined with Unx in rats, it has been found to be suitable for studying DN caused by T2D^14^. B6 mice that receive a 60 energy% (EN%) HFD display albuminuria, glomerular lesions and increased extracellular matrix deposition (ECM)^15^. In most T2D studies, HFD commonly contains 50–60 EN% fat, which is considerably more than the average human fat intake in Europe (i.e. 28.5–46.2 EN%)^16^. In contrast to humans, mice are herbivores meaning that they are less adapted to tolerate high and even moderate amounts of fat compared to humans. Therefore, the excessive fat intake of mice in HFD studies is often not representative of the human pathophysiology of obesity-related diseases. Only a limited number of studies have used a more realistic 43 EN% HFD as a T2D model^17^. The aim of our study is to identify the most effective method to induce DN in type 1 and type 2 diabetes models in the relatively resistant B6 strain.

To study a T1D-induced nephropathy model, mice were fed a control diet combined with a multiple low-dose STZ treatment alone or in combination with UNx. In addition, mice were fed a western diet in combination with a multiple low-dose STZ treatment alone and in combination with UNx to study the most effective model for T2D-induced nephropathy.

## Results

Mice were randomly assigned to seven groups: control diet group (C), control diet+STZ (CS), control diet+STZ+UNx, western diet (W), western diet+STZ (WS), western diet+STZ+UNx (WSU) or western diet+UNx (WU). The abbreviations referring to the study groups are shown in Table 1.

**Table 1:** Description of the groups.

Abbreviation	Diet	Injections	Surgery
C	Control	Vehicle	Sham
CS	Control	STZ	Sham
CSU	Control	STZ	UNx
W	Western	Vehicle	Sham
WS	Western	STZ	Sham
WSU	Western	STZ	UNx
WU	Western	Vehicle	UNx

### Bodyweight, food and water intake, fasting blood glucose, fasting plasma insulin, HOMA-IR, HOMA-beta

Bodyweight was lower in all STZ-treated mice compared to their respective control groups (C, or W and WU), except for W vs. WS. Food and water intake were higher in CS and CSU mice compared to C mice. In the western diet groups (W, WS, WSU, WU) only WS mice show higher food and water intake. Although the differences in food and water intake seemed clear, the limited number of data points of these parameters (as food and water intake were determined per cage and not per animal) did not allow for statistical testing. Fasting blood glucose was increased and fasting plasma insulin was decreased in all STZ-treated mice. HOMA-IR was equal between all mice. HOMA-beta was dramatically decreased in STZ-treated groups. All parameters are shown in Table 2.

**Table 2:** Group characteristics and biochemical parameters.

	C	CS	CSU	W	WS	WSU	WU
BW, baseline (g)	22.1 (1.6)	21.7 (0.9)	22.7 (1.1)	21.8 (1.2)	21.5 (1.6)	21.1 (1.1)	22.1 (1.3)
BW, endpoint (g)	30.2 (1.8)	25.2 (2.2)^a^	21.1 (1.9)^b^	31.6 (2.3)	27.8 (2.1)^e^	26.7 (1.7)^a,e^	32.2 (4.3)
Food intake (g/day)	3.1 (0.17)^NT^	3.8 (0.5)^NT^	3.6 (0.4)^NT^	3.6 (0.3)^NT^	4.0 (0.4)^NT^	3.4 (0.4)^NT^	3.5 (0.5)^NT^
Caloric intake (kcal/day)	10.8 (0.7)^NT^	13.2 (1.8)^NT^	12.6 (1.5)^NT^	15.8 (1.4)^NT^	17.9 (2.0)^NT^	15.1 (1.7)^NT^	15.9 (2.3)^NT^
Water intake (g/day)	3.9 (1.3)^NT^	19.3 (3.5)^NT^	22.9 (2.3)^NT^	4.7 (1.5)^NT^	8.4 (4.5)^NT^	5.4 (1.3)^NT^	1.6 (0.9)^NT^
Urinary volume (ml/day)	1.1 (0.4)	11.0 (12.0)	27.3 (6.0)^c^	0.5 (0.3)	1.2 (1.3)	3.6 (2.9)^a^	1.0 (0.7)
Plasma creatinine (uM)	10.3 (3.4)	10.4 (2.0)	11.0 (3.5)	7.9 (2.5)	8.1 (3.4)	10.6 (1.9)	10.5 (2.6)
Plasma Urea (mM)	8.5 (2.4)	7.5 (0.9)^g^	12.2 (3.1)^a^	8.0 (1.3)	8.1 (0.6)	9.1 (1.3)	9.0 (0.8)
FBG (mM)	9.7 (1.4)	22.9 (6.3)^b^	23.5 (3.5)^c^	9.9 (1.5)	20.5 (4.0)^b,e^	19.3 (9.9)	10.1 (2.4)
FPI (uU/mL)	26.9 (8.4)	12.8 (2.3)^b^	11.3 (1.4)^c^	29.8 (15.7)	12.0 (5.4)^a^	12.0 (2.7)^a,d^	21.7 (5.8)
HOMA-IR	11.3 (3.5)	12.8 (3.4)	12.3 (1.2)	12.9 (7.0)	10.6 (3.1)	8.4 (3.1)	9.8 (3.3)
HOMA-beta	87.5 (35)	16.7 (4.9)^a^	11.3 (3.4)^b^	84.4 (44)	15.1 (8.6)^c,f^	20.1 (9.9)^b,d^	69.6 (28)
Systolic blood pressure (mmHG)	171 (46)	166 (28)	143 (45)	152 (43)	154 (43)	161 (26)	184 (34)

Values are expressed as mean (SD). ^a^p < 0.05,^b^p < 0.01 and ^c^p < 0.0001 vs its diet control (C or W); ^d^p < 0.05, ^e^p < 0.01 ^f^p < 0.0001 vs WU; ^g^p < 0.01 vs CSU. FBG = fasting blood glucose, FPI = fasting plasma insulin, ^NT^not tested.

### Systolic blood pressure and urinary and renal function parameters

**Figure 1:**
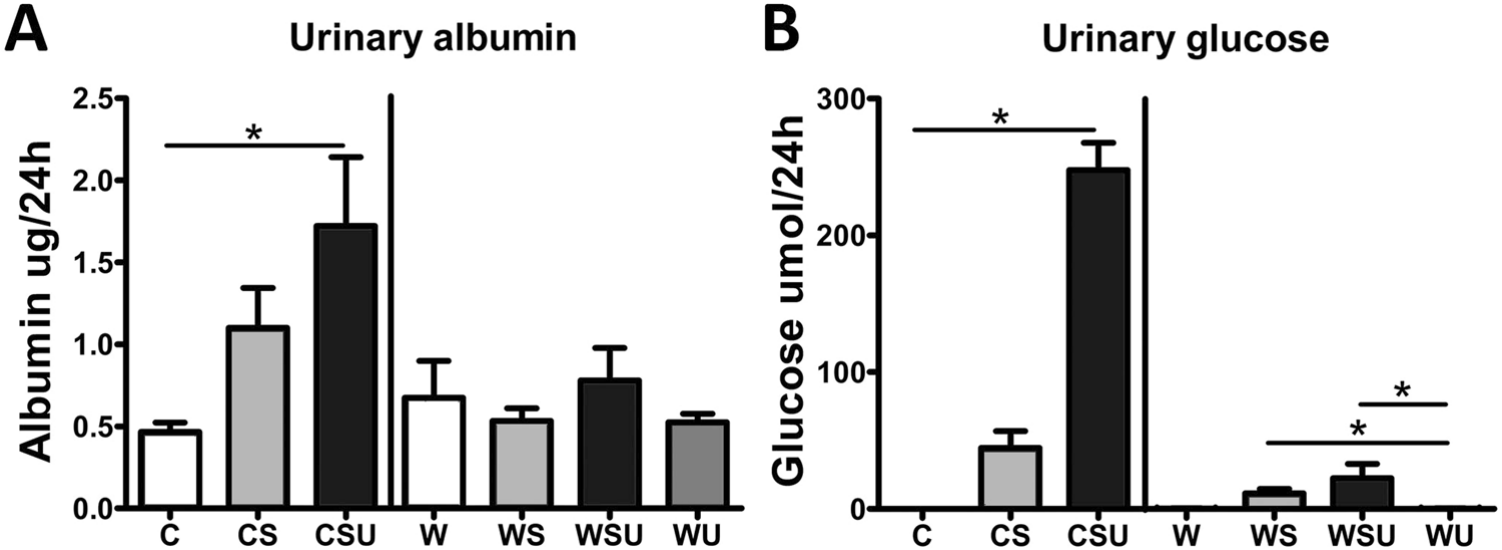
Albumin and glucose excretion in urine. (**A**) Albumin excretion in 24 hours urine was measured by ELISA. CSU mice showed increased urinary albumin levels. (**B**) Glucose was measured by an enzymatic glucose kit. CSU mice showed a large increase in urinary glucose compared to C, and WS and WSU mice had increased urinary glucose excretion compared to WU mice. Data are represented as mean ± SEM. *p < 0.05.

Polyuria was observed in both CSU and WSU compared to their diet controls. Although CS and WS mice showed higher urinary volume as well, this increase was not significant due to the large variation. Both systolic blood pressure and plasma creatinine in CS, CSU, WU and WSU were unaffected compared to the control groups. Plasma urea levels were only increased in CSU mice compared to C mice. All above parameters are shown in Table 2. Urinary albumin levels (Fig. 1A) per 24 h were only significantly increased in CSU mice compared to C animals and urinary albumin levels were not changed in WS, WSU and WU mice compared to W mice. Urinary glucose levels (Fig. 1B) per 24 h were dramatically increased in CSU mice, while this increase was absent in CS mice. In the western diet groups, increases in urinary glucose levels were less prominent, but significantly increased in WS and WSU mice compared to WU.

### Glomerular lesions

**Figure 2:**
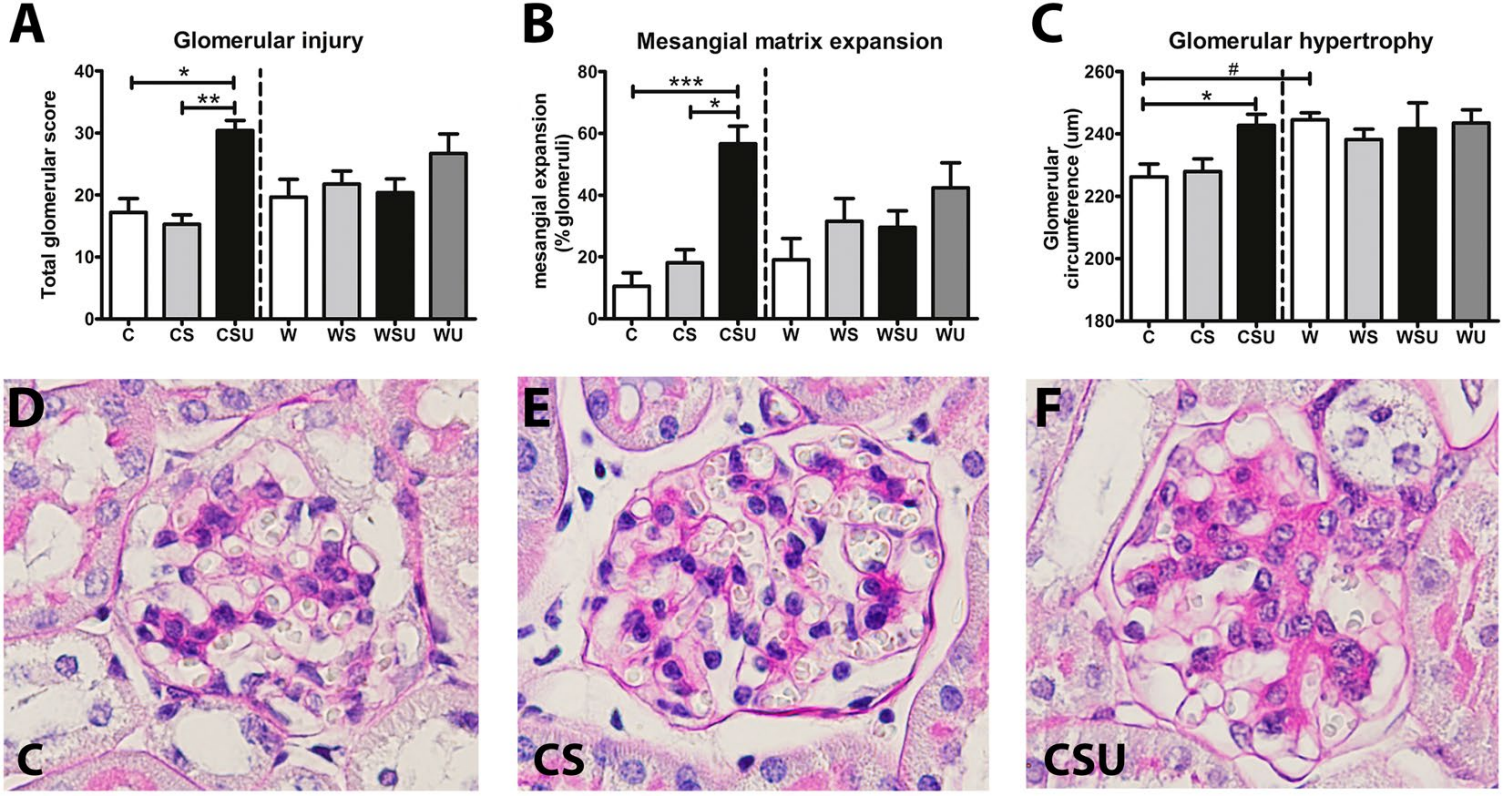
Glomerular lesions on PAS-D stainings. (**A**) Glomerular injury score was calculated by the sum of mesangial proliferation, mesangial matrix expansion and mesangiolysis. (**B**) Mesangial matrix expansion was scored as absent or present and results are shown as the percentage of glomeruli that showed mesangial matrix expansion. (**C**) The circumference of glomeruli was determined as a measure for glomerular hypertrophy. CSU mice show increased glomerular injury, mesangial expansion and glomerular hypertrophy. W mice show elevated glomerular hypertrophy when compared to C mice. Representative photographs (magnification 40x) of the PAS-D stainings are shown for C mice (**D**), CS mice (**E**), and CSU mice (**F**). Data are represented as mean ± SEM. *p < 0.05, **p < 0.01, ***p < 0.0001, ^#^p < 0.0001 in C vs. W comparison.

**Figure 3:**
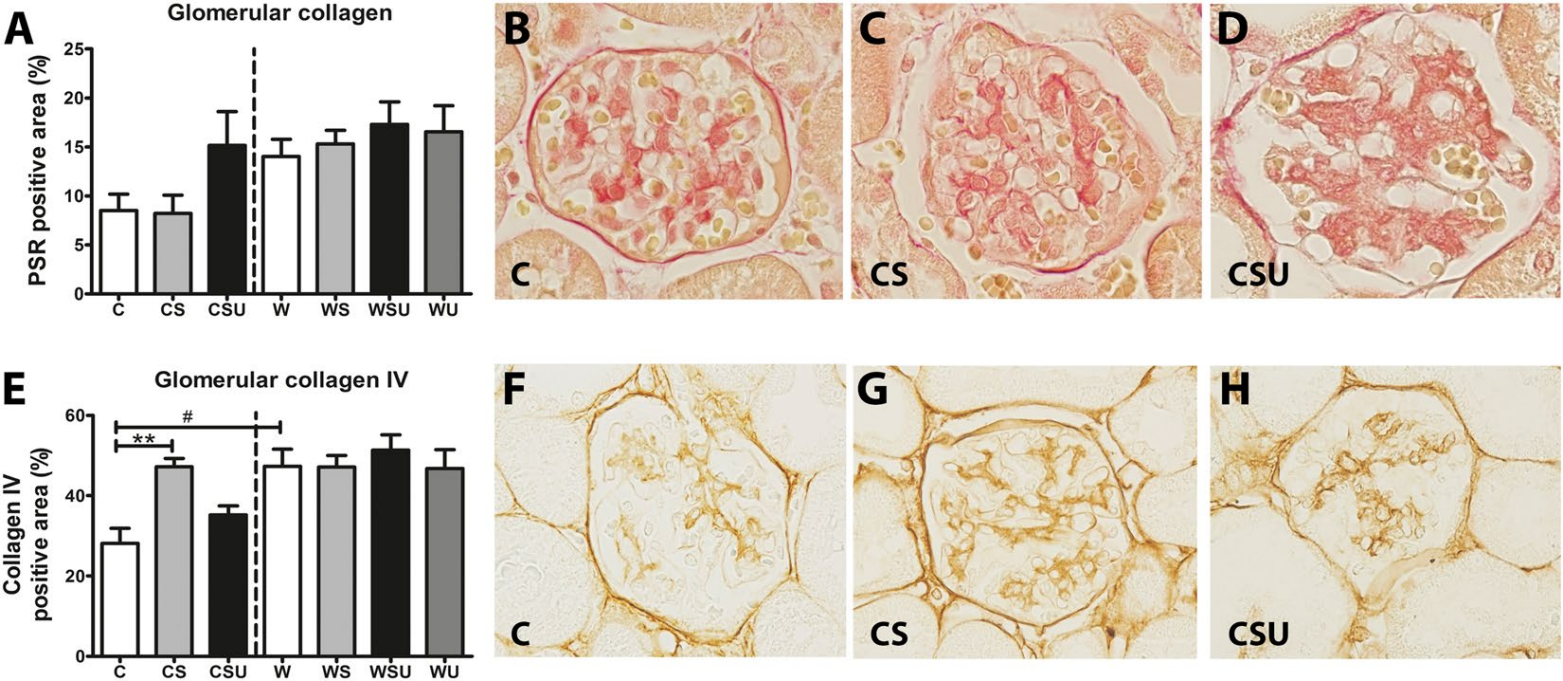
Glomerular collagen expression. (**A**) Quantification of glomerular collagen by a picrosirius red (PSR) staining. No significant differences were found between the groups, however, CSU and all western diet groups tend to have increased collagen deposition in the glomeruli. (**B**–**D**) Representative photographs (magnification 80x) of glomerular PSR-positive area in C, CS, and CSU mice, respectively. (**E**) Quantification of glomerular immunostaining of collagen IV. CS mice have elevated glomerular collagen IV deposition and also W mice show increased collagen IV compared to C mice. (**F**–**H**) Representative photographs (magnification 80x) of collagen IV staining from C, CS, and CSU mice respectively. Data are represented as mean ± SEM. **p < 0.01, ^#^p < 0.05 in C vs. W comparison.

The total glomerular injury score (Fig. 2A) was higher in CSU mice, compared to C mice. Similarly, the percentage of glomeruli showing mesangial matrix expansion (Fig. 2B) was only increased in CSU animals. See Supplementary Figure S1 for representative photographs of mesangial matrix expansion in W, WS, WSU and W mice. Glomerular hypertrophy (Fig. 2C) was observed in CSU mice only, however, W mice also showed enlarged glomeruli compared to C mice. Mesangiolysis was observed in some glomeruli, however, the frequency was similar between groups (data not shown). Quantification of the PSR-stained kidney sections (Fig. 3A–D) revealed a tendency towards increased glomerular collagen in CSU mice, while collagen IV (Fig. 3E–H) was only increased in CS animals. Glomerular collagen and collagen IV in kidneys of the western diet fed mice were not affected by STZ treatment, UNx or both. Nevertheless, collagen IV was significantly higher in W mice compared to C mice. See Supplementary Figure S2 for representative photographs of glomerular collagen and collagen IV stainings in W, WS, WSU and W mice. These data suggest that the increased glomerular hypertrophy and collagen IV deposition are solely the effect of the western diet, and therefore independent of diabetes and UNx.

### mRNA expression of fibrosis markers

**Figure 4:**
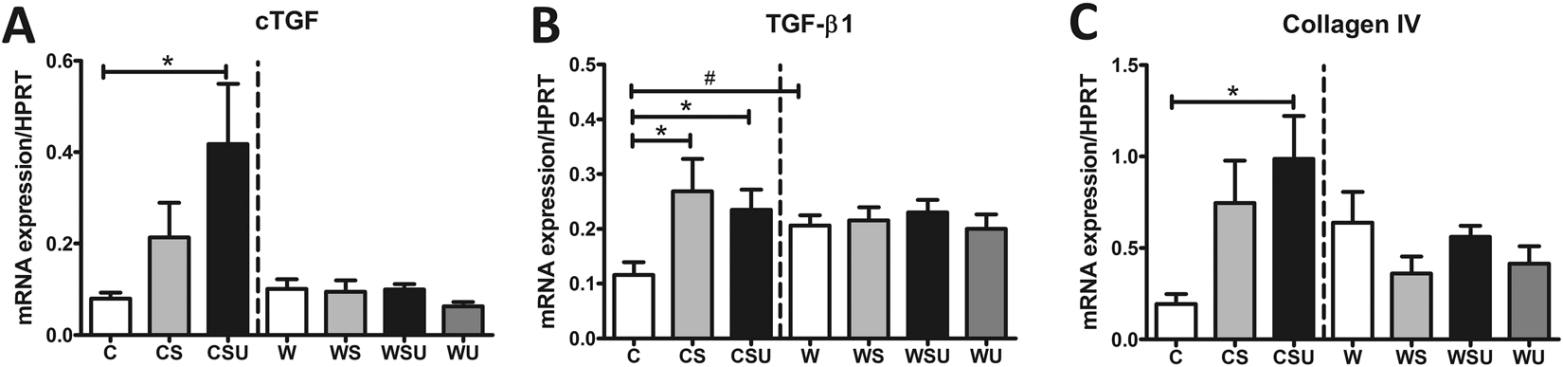
Renal mRNA expression. mRNA expression in renal tissue was determined by qPCR. (**A**) Connective tissue growth factor (cTGF) was upregulated in the CSU mice. (**B**) Transforming growth factor-β1 (TGF-β1) was upregulated in CS and CSU mice, but also in W mice compared to C mice. (**C**) Collagen IV was upregulated in CSU mice only. Data are represented as mean ± SEM *p < 0.05, ^#^p < 0.05 in C vs. W comparison.

TGF-β1 (Fig. 4B) was upregulated in both CS and CSU mice. cTGF and collagen IV (Fig. 4A,C) were upregulated in CSU mice only. Differential expression of fibrosis markers was not observed in western diet groups (Fig. 4A–C). Nevertheless, W mice showed significantly higher TGF-β1 expression compared to C mice, which was not further enhanced by diabetes or UNx, suggesting that a western diet alone is pro-fibrotic.

### Tubular injury and tubulo-interstitial fibrosis

**Figure 5:**
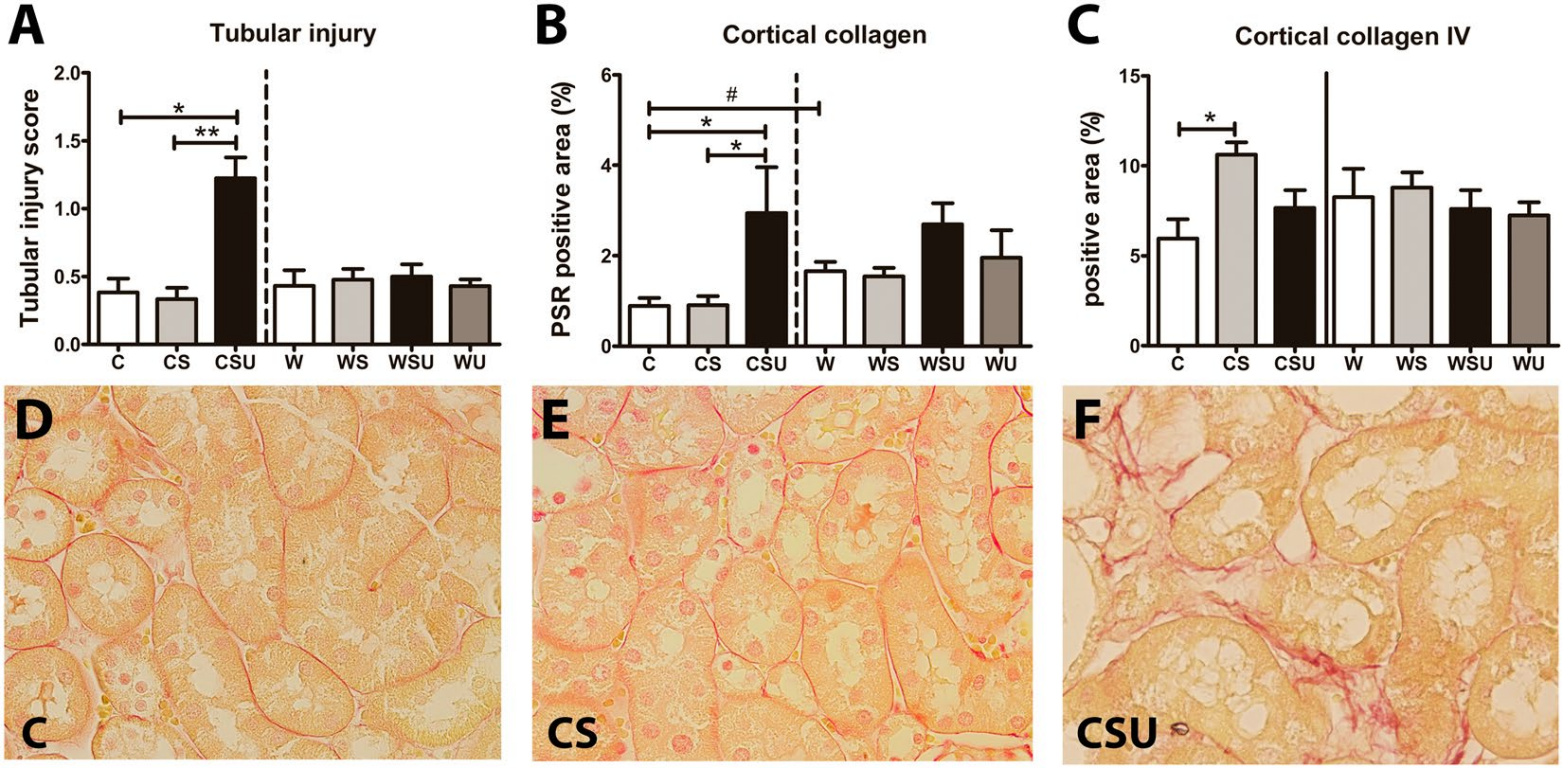
Tubular injury score and cortical collagen expression. (**A**) Tubular injury was scored based on tubular dilatation and atrophy, and the presence of the brush border. Tubular injury was increased in CSU mice. (**B**) Cortical collagen expression was determined in picrosirius red (PSR)-stained renal sections. Tubulo-interstitial collagen deposition was elevated in both CSU mice and W mice. (**C**) Cortical collagen IV was quantified in immunostained sections. CS mice showed increased collagen IV deposition in the tubulo-interstitial area. (**D**–**F**) Representative photographs (magnification 40x) of cortical PSR sections in C, CS, and CSU mice, respectively. Data are represented as mean ± SEM. *p < 0.05, **p < 0.01, ^#^p < 0.01 in C vs. W comparison.

Tubular injury score (Fig. 5A) was significantly higher in CSU kidneys compared to C and CS mice. Collagen positive area in the cortex (Fig. 5B) was increased in CSU as well, but collagen IV (Fig. 5C) was increased in CS only. Also, W mice show higher cortical collagen deposition compared to C mice. See Supplementary Figure S3 for representative photographs of cortical collagen deposition in W, WS, WSU and W mice.

### GBM

**Figure 6:**
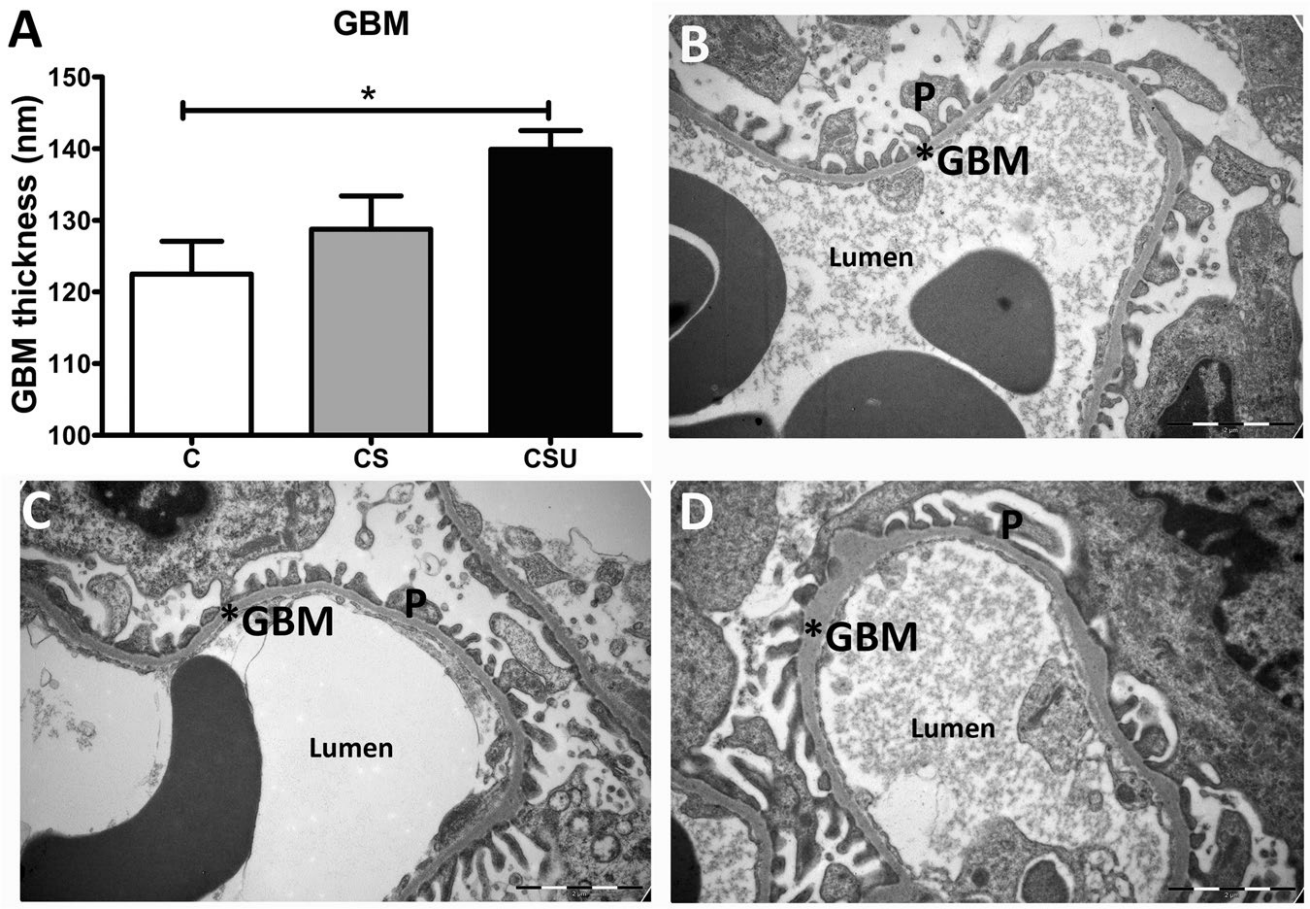
GBM thickness. (**A**) Glomerular basement membrane (GBM) thickness was measured by electron microscopy. CSU mice showed increased GBM thickness compared to C mice. (**B**–**D**) Representative EM photographs (magnification 18500x) of C, CS and CSU mouse kidney, respectively. Data are represented as mean ± SEM and the scale bar shows 2 μm. *p < 0.05, P = podocyte.

Since STZ and STZ+UNx in western diet fed mice did not show signs of DN, GBM thickness was determined in control diet groups only. Thickening of the GBM was significantly elevated in CSU mice, but no thickening was observed in CS glomeruli (Fig. 6A–D).

## Discussion

B6 mice are considered to be relatively resistant to the development of STZ-induced DN. In this study we aimed to identify the most effective method to induce DN in a T1D and T2D model in the relatively resistant B6 strain. We demonstrated that combining STZ and UNx is the most effective way to induce DN in a T1D model in B6 mice. This model induced albuminuria, thickening of the GBM, mesangial matrix expansion, glomerular hypertrophy, early glomerulosclerosis, upregulation of pro-fibrotic markers, tubular injury and tubulo-interstitial fibrosis. Most of these hallmarks were less pronounced or absent in mice that received STZ only, although the CS mice had more glomerular and total cortical collagen IV expression, and higher levels of TGF-β1 mRNA than controls. In contrast, our study found only minor effects of a 15-weeks western diet alone, however, a western diet in combination with STZ, or in combination with STZ and UNx, was not effective in inducing significant albuminuria, glomerular and tubular injury.

In the T1D-induced DN model, we compared the most commonly used multiple low-dose STZ model with a combination model of multiple low-dose STZ and UNx. We found differences in glomerular and tubulo-interstitial ECM composition between CS and CSU. We showed that CS merely have increased collagen IV deposits, while CSU glomeruli have increased PSR positive tissue (PSR stains collagen I and III fibres^18^), but not collagen IV. We propose that this difference may be explained by the different triggers of ECM production in CS and CSU kidneys, as renal hemodynamics are changed directly after UNx. In CS mice the major contributor to renal injury is hyperglycaemia, while in CSU mice both hyperfiltration and hyperglycaemia are major contributors to the renal injury. Indeed, STZ treatment in itself can lead to hyperfiltration^8^, however, hyperfiltration will gradually develop after initiation of hyperglycaemia, whereas hyperfiltration in CSU mice starts directly after UNx and persists for the duration of the study period. Hyperfiltration, which is one of the first events to be observed in DN, can induce mechanical stretching which stimulates glomerular hypertrophy and ECM production in response to mesangial cell injury^19^. Hyperfiltration eventually results in excessive ECM proteins pooling in the mesangial area of the glomeruli^20^, however, this does not explain the difference in ECM composition. A major mediator in the hypertrophic mechanism of hyperfiltration is the renin-angiotensin system (RAS). The RAS is known for its role in blood pressure regulation and sodium/potassium balance, it also stimulates the production of pro-fibrotic cytokines and reactive oxygen species^20^. Hyper activation of RAS in local tissue is thought to contribute to progressive renal injury in subtotal nephrectomy models^21,22^. Not only do indirect effects of hypertension account for this effect, but the direct fibrogenic effect of angiotensin II also plays a significant role^23,24^.

It has been described that mesangial cells respond differently to stimuli, such as glucose, insulin and angiotensin II, in terms of ECM protein production^23^. Hyperglycaemia induces deposition of basically all ECM proteins, but primarily fibronectin, laminin and collagens IV and VI^23,25^. On the contrary, angiotensin II enhances the production of collagen I^26^, but not collagen IV^27^. Angiotensin II is found to be elevated in DN, through the direct upregulation of glucose^28^, however, it is unclear how UNx affects angiotensin II levels. Nevertheless, intrarenal hypertension and subsequent glomerular hyperfiltration is found to increase angiotensin II levels shortly after hypertension induction^29^. As hyperfiltration is a major consequence of UNx, we suggest that angiotensin II levels are elevated in UNx, which may explain the differences in ECM composition between CS and CSU mice from this study. Unfortunately, our specific study design did not allow for reliable determination of intrarenal angiotensin II levels.

In addition to glomerular lesions, our study showed increased injury and fibrosis in the tubular compartment of CSU mice. In current publications on DN, the development of tubular atrophy, loss of brush border and tubular dilatation are often absent or not described, and the same is true for tubulo-interstitial fibrosis and GBM thickening^8,30,31^. The UNx that was applied in this model has demonstrated the ability to induce these pathologies in the GBM and tubular compartment, which is an essential advantage of this model. Besides proteinuria, we suggest that this tubular damage in CSU mice is a result of glucotoxicity in the tubuli. In a non-diabetic situation, excessive blood glucose that is filtered in the glomeruli can be reabsorbed by sodium-glucose linked transporter 2 (SGLT2) in the proximal tubules. During diabetes the amount of filtered glucose is abundant and the SGLT2 are saturated with the resulting glycosuria. In diabetic unilateral nephrectomised mice, all the glucose is excreted via the remaining kidney, resulting in a more rapid SGLT2 saturation. Exposure of tubular epithelial cells to high concentrations of glucose has been shown to induce the production of reactive oxygen species (ROS) which in turn induces epithelial-to-mesenchymal transition (EMT)^32^. This is an important process in DN progression and contributes to tubulo-interstitial fibrosis.

In contrast to the control diet mice, glomerular lesions, albuminuria and tubular damage were not affected by STZ or STZ and UNx in mice that were fed a western diet, despite the presence of hyperglycaemia. Nevertheless, we demonstrated that a 15-week western diet alone induced glomerular hypertrophy, enhanced glomerular and cortical collagen deposition, and upregulation of TGF-β1 mRNA expression, despite a very mild metabolic syndrome phenotype.

These findings contrast with previous studies, where B6 mice on a HFD develop albuminuria, glomerular lesions and increased extracellular matrix deposition (ECM)^15^. However this difference is likely due to the differences in fat content, as most HFD contain up to 60 EN% fat, compared to the 43% in our western diet. On the other hand, Glastras *et al*. also utilized a 43% fat diet to induce T2D, both with and without the administration of a single high dose of STZ. They observed a mild increase in the albumin-to-creatinine ratio and serum creatinine, and a profound increase in glomerulosclerosis and tubulo-interstitial fibrosis in high fat diet mice^17^. A key difference between the article by of Glastras *et al*. and our study is the diet intervention period of 29 weeks versus 15 weeks, respectively. In our study, the 15-week western diet was not sufficient to induce a metabolic syndrome phenotype with the accompanying renal injury.

In addition to the non-genetic models of DN, the leptin receptor deficient db/db mice, on a B6 background, also develop renal damage^33^, which can be further accelerated by UNx^34^.

Our department previously induced renal injury in mice using a 16-week western diet intervention^35,36^. Wildtype B6 mice did not increase in bodyweight, whereas the amount of adipose tissue and HOMA-IR were elevated in western diet fed mice. In addition, mRNA expression of fibrosis markers were upregulated and cholesterol and phospholipids accumulated in the kidneys of western diet fed mice as well. Previous studies show that a high cholesterol diet induces fibrosis in the liver^37,38^. Our previous work shows that this holds true for renal fibrosis as well^35^, which may explain the increased collagen deposition in the cortex and glomeruli, glomerular hypertrophy, and upregulation of TGF-β1 mRNA expression we observed in the present study. Bakker *et al*. did not study this phenomenon in the glomerular compartment, however, other studies also observed hypertrophic and fibrotic effects of a high cholesterol diet in the glomeruli^39,40^.

The western diet itself had a pro-fibrotic effect in all western diet-fed groups, nonetheless, western diet seems to be protective against injury caused by STZ, UNx or the combination of STZ+UNx, as no significant differences were found between these groups in regard to the urinary albumin excretion, glomerular injury, tubular injury, and expression of pro-fibrotic genes. Although these findings are in contrast with previous studies, where renal injury induced by a HFD (50–60%) was augmented by combining HFD with UNx^41^ or a low-dose STZ treatment^42^, Glastras *et al*. did not show additional renal injury as a result of STZ in mice on a HFD (43%). We propose that the protective effect in our study may be explained by metabolic changes in the renal cells. In hyperfunctioning nephrons, e.g. in the remnant kidney after UNx and to a lesser extent diabetic kidneys, the energy demand of nephrons exceeds the available metabolic substrate, which was reported to result in hypoxia/ischemia, acidosis and ROS production^20^. As tissue energy levels decrease due to insufficient production of insulin, we propose that the increased energy requirement of the hyperfiltrating kidney in CSU mice cannot be met, resulting in renal injury. Diabetic mice on a western diet are likely to be less affected by the reduced glucose uptake capacity, as they have more energy available from lipids. Thus, we suggest that western diet fed mice have more energy available to provide the hyperfunctioning nephron with sufficient energy to avoid hypoxia/ischemia-induced injury.

On the contrary, Taneja *et al*. show elevated glomerular injury in STZ-induced diabetic mice on a high cholesterol diet, compared to a control diet^39^. However, these results were observed after 36 weeks of intervention. This difference suggests that a western diet and/or high cholesterol diet could be protective in the initial stage (<4 months), but become harmful when the diet continues (i.e. 9 months). It is well described that a HFD with STZ treatment is effective in DN induction^43^, indicating that a HFD is preferred over a western diet as a model of T2D-induced nephropathy.

In addition to the renal fibrosis markers, urinary glucose excretion was increased in WS and WSU mice, however, this glucose excretion was only a fraction of the glucose excretion observed in the control diet mice. This difference must be caused by either blood glucose levels differences, glucose reuptake differences, or both. Diabetic mice on a western diet indeed have lower plasma glucose levels than diabetic control diet fed mice, possibly a mere reflection of the lower carbohydrate content of the western diet. In addition, recent findings from Chichger *et al*. indicate that a western diet changes the expression of the glucose transporters (GLUT2, SGLT1 and 2) on the proximal tubule brush border membrane. As a result, western diet increases the glucose resorption from the pre-urine^44^. The combination of lower blood glucose concentrations and possibly increased glucose resorption might explain the lower urinary glucose levels in these diabetic western diet fed mice.

Limitations of our study include the absence of a control diet group with UNx only, which would provide better insight into the contribution of UNx under normoglycaemic conditions. However, several studies have shown that UNx in the absence of diabetes does not result in features of DN, except renal hypertrophy^11,12^. Therefore, we assume that UNx only has a limited effect on the glomerular and tubular lesions demonstrated in the CSU mice. Also, despite the effective induction of DN by combining STZ and UNx, this model does not completely comply with the guidelines for DN models from the AMDCC^4^. Extension of the diabetes period by 2–6 weeks would be recommended when a more pronounced DN phenotype is desired. Previous studies performed in B6 mice that were subjected to STZ and UNx show albuminuria and increased mesangial matrix deposition at earlier time points^30,45,46^. However, it is striking that the STZ dose varies from 40–60 mg/kg between studies. Also, sensitivity to STZ appears to be dependent on the animal supplier^47^. These two factors may partially explain the differences in DN phenotype between studies. However, we think that the major differences in DN phenotype can be attributed to differences in the microenvironment of the research institutes, which has been demonstrated after gut microbiota depletion^48^ and supplementation of pro-biotics^49^.

This study showed that control diet fed mice that were subjected to both multiple low-dose of STZ and UNx is most effective in inducing T1D-induced nephropathy in the relatively resistant B6 mice. In addition, a western diet combined with STZ alone or in combination with UNx was not found to effectively induce renal injury in our study.

## Materials and Methods

### Animals

Five-week-old male C57BL6/J mice (18–22 grams) were purchased from Charles Rivers. Mice were housed in IVC cages, with 12/12 light/dark cycle, receiving food and water ad libitum. Seventy-three mice were randomly assigned to seven groups (n = 8–15 per group, 3–4 mice per cage): control diet group (C), control diet+STZ (CS), control diet+STZ+UNx (CSU), western diet (W), western diet+STZ (WS), western diet+STZ+UNx (WSU) or western diet+UNx (WU). Directly after arrival mice were fed either a control diet or a western diet for 15 weeks. Control diet contains 19 EN% protein, 70 EN% carbohydrates and 11 EN% fat. Western diet contains 17 EN% protein, 40 EN% carbohydrates and 43 EN% fat, with additional 0,15% cholesterol. These diets were obtained from Arie Blok Animal Nutrition (Woerden, the Netherlands) as previously described^35,36^. This mouse experiment was approved by the Animal Care and use Committee of the University of Amsterdam.

### Experimental procedures

One week after arrival, nephrectomy of the left kidney was performed in isoflurane anesthetized mice (3–4% induction, 1.5–2.5% maintenance, 100% oxygen). All mice received preoperative (0.5 h) and postoperative analgesia (6 h and 20 h) via subcutaneous injection of 0.1 mg/kg buprenorphine (Temgesic, Schering-Plough, Maarssen, the Netherlands). Mice were allowed to recover for one week, followed by freshly dissolved 50 mg/kg intraperitoneal streptozotocin (Sigma-Aldrich, Zwijndrecht, the Netherlands) or 100 ul citrate injections for 5 consecutive days. Bodyweight, food and water intake, and blood glucose levels (Contour next, Ascensia diabetes Care, Parsippany, NJ, USA) were weekly monitored and blood samples were collected weekly via saphenous vein puncture in heparinized Microvette collection tubes (Sarstedt, Nümbrecht, Germany). The intake of food and water was determined per cage and the intake per day per mouse was calculated.

Twelve weeks after the last STZ injection 24-hour urine was collected using metabolic cages. One week later, mice were anesthetized with FMA (0.47 mg/kg fentanyl, 9.38 mg/kg midazolam, 9.38 mg/kg acepromacin, via IP injection), systolic blood pressure was measured with the tail-cuff method and mice were killed by exsanguination via eye extraction, followed by cervical dislocation. The right kidney, epididymal white adipose tissue, pancreas, liver and spleen were cut in two parts and were either fixed in 10% formalin or snap-frozen in liquid nitrogen. All blood and urine samples were centrifuged at 1550xg for 20 minutes at 4 °C, and all supernatants were stored at −80 °C until analysis.

Mice that lost >10% of their bodyweight in one week, due to severe hyperglycaemia, received 1/2 Linbit insulin pellet (LinShin, Toronto, Canada). A total of six mice received insulin (CS n = 2; CSU n = 4). All insulin-treated mice remained hyperglycaemic. Conversely, mice that did not reach blood glucose levels >16 mM within 2 weeks after STZ injections were considered as non-responders and were excluded from all analyses (CSU n = 3; WSU n = 4). Other reasons to prematurely sacrifice mice or to exclude them from analysis were severe skin wounds (W n = 1; WS n = 1), extreme hyperglycaemia (>33,3 mM) with weight loss (>10%) before insulin could be administered (CSU n = 2) and signs of a cystic kidney (C n = 2). All experimental procedures were performed in compliance with the Dutch government guidelines.

### Biochemical analysis

Plasma creatinine and plasma urea were measured using standardized clinical diagnostic protocols. Albumin levels in 24-hour urine were measured by means of sandwich ELISA using the goat anti-mouse albumin antibody (Bethyl Laboratories, Montgomery, TX, USA). Urinary glucose concentrations were determined in deproteinized samples (PCA kit, Biovision, Milpitas, CA, USA) by a glucose assay kit (Abcam, Cambridge, UK) and fasting insulin levels were determined by ultrasensitive mouse insulin ELISA kit (CrystalChem, Downers Grove, USA). Insulin resistance was estimated with the homeostatic model assessment- insulin resistance (HOMA-IR) using the following formula: HOMA-IR = [fasting plasma insulin(µU/mL)*fasting blood glucose (mmol/L)/22.5]^50,51^. HOMA-beta was calculated as follows: HOMA-beta = (20*fasting plasma insulin)/(fasting blood glucose-3,5)^51^.

### Histology

Renal tissues were fixed in 10% formalin for 24 hours and embedded in paraffin. Four micrometer sections were cut for all stainings. For examining glomerular lesions and tubular injury, slides were stained with Periodic acid–Schiff reagents after diastase digestion (PAS-D), and scored by a pathologist, semi-quantitatively, in a blinded fashion. 25 glomeruli were scored for the presence of mesangial matrix expansion (0 = absent; 1 = present), mesangiolysis (0 = absent; 1 = present) and mesangial proliferation (>3 cells per mesangial area). A total glomerular lesion score was calculated by the sum of mesangial matrix expansion, mesangiolysis and mesangial proliferation. Tubular injury was scored in the cortical region on a scale from 0 to 4 (0 = 0%, 1 = 0–25%, 2 = 25–50%, 3 = 50–75% 4 = 75–100% injured). This score was based on tubular dilatation, loss of the brush border and tubular atrophy. Picrosirius Red (PSR)-stained slides were digitalized using the Philips scanner, followed by quantification of the cortical collagen in three non-overlapping areas of the cortex. Glomerular hypertrophy was determined by measuring the mean glomerular circumference of the 20 largest glomeruli. Four large fields were selected on scanned PAS-D slides and the five largest glomeruli per field were selected for this measurement. PSR and PAS-D stained slides were scanned using the Digital Pathology solutions software (Philips, the Netherlands). Cortical collagen IV (goat-anti-collagen IV, Merck Milipore, Amsterdam, the Netherlands) positive areas were determined in 10 high power fields of digital images. Glomerular PSR and Collagen IV were quantified in 15–20 glomeruli. All staining quantifications were performed using image analysis using Fiji software (ImageJ, https://fiji.sc/).

### Electron microscopy

Cortical right kidney samples were fixed in Karnovsky’s fixative until analysis. Samples were further prepared as previously described^52^. Photographs were taken with the Fei Tecnai-12 transmission electron microscope (FEI, Eindhoven, the Netherlands) with a magnification of 18500x. Five random photographs were acquired per glomerulus and the GBM thickness was assessed by measuring five random and unbiased points in each photograph.

### Quantitative real-time PCR

Total RNA was isolated from frozen kidney sections, using Tri-reagent (Sigma Aldrich) according the manufacture’s protocol. cDNA was generated using oligo-dt primers. *cTGF*, *TGF-β1* and *Collagen IV* mRNA expression was measured by Roche Light Cycler 480 (Roche, Woerden, the Netherlands) with SYBR green PCR master mix (Bioline, Ranst, Belgium). Gene expression was normalized against *HPRT* gene expression and analysed with linear regression analysis. Primer sequences: cTGF-F: TGACCTGGAGGAAAACATTAAGA; cTFG-R: AGCCCTGTATGTCTTCACACTG; TGF-*β*1-F: GCAACATGTGGAACTCTACCAGAA; TGF-*β*1-R: GACGTCAAAAGACAGCCACTCA; Collagen IV-F: CTGGAGAAAAGGGCCAGAT; Collagen IV-R: TCCTTAACTTGTGCCTGTCCA; HPRT-F: TGTCCGTCGTGGATCTGAC; HPRT-R: CCTGCTTCACCACCTTCTTG.

### Statistical analysis

Normally distributed parameters (tested with D’Agostino & Pearson omnibus normality test) were tested for statistical significance with One-way ANOVA and not normally distributed parameters with Kruskal-Wallis testing. Bonferroni correction was applied to correct for multiple testing. To test the effect of the western diet, we compared C and W groups with the Mann-Whitney test. Statistical tests were performed separately for the control and western diet groups. Food, caloric and water intake was determined per cage (2–3 cages per group), therefore no statistical test was performed on these parameters. Results are reported as mean ± SEM, unless indicated otherwise. P values < 0.05 were considered as statistically significant.

### Data availability statement

The datasets generated and analysed during the current study are available from the corresponding author on reasonable request.

## Electronic supplementary material

Supplementary Information
