# Author Correction: Combining streptozotocin and unilateral nephrectomy is an effective method for inducing experimental diabetic nephropathy in the ‘resistant’ C57Bl/6J mouse strain

**DOI:** 10.1038/s41598-018-38075-4

**Published:** 2019-02-27

**Authors:** Melissa Uil, Angelique M. L. Scantlebery, Loes M. Butter, Per W. B. Larsen, Onno J. de Boer, Jaklien C. Leemans, Sandrine Florquin, Joris J. T. H. Roelofs

**Affiliations:** Department of Pathology, Academic Medical Center, University of Amsterdam, Amsterdam, The Netherlands

Correction to: *Scientific Reports* 10.1038/s41598-018-23839-9, published online 03 April 2018

In Fig. 1A, the y-axis has the incorrect scale. The correct Figure 1 appears below.

**Figure 1:**
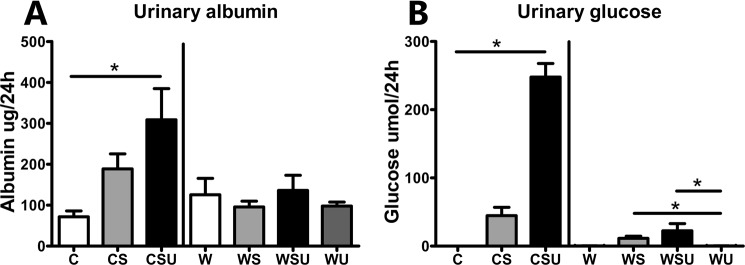
Albumin and glucose excretion in urine. (**A**) Albumin excretion in 24 hours urine was measured by ELISA. CSU mice showed increased urinary albumin levels. (**B**) Glucose was measured by an enzymatic glucose kit. CSU mice showed a large increase in urinary glucose compared to C, and WS and WSU mice had increased urinary glucose excretion compared to WU mice. Data are represented as mean ± SEM. *p < 0.05.

