# Supplementary material for: Combining streptozotocin and unilateral nephrectomy is an effective method for inducing experimental diabetic nephropathy in the ‘resistant’ C57Bl/6J mouse strain
Source: Sci Rep. 2018 Apr 3;8:5542. doi: 10.1038/s41598-018-23839-9 (PMC5882654; doi:10.1038/s41598-018-23839-9)
Supplement: Supplementary file 1 — Supplementary Information [file 41598_2018_23839_MOESM1_ESM.doc]

**Supplementary Information**

**Combining streptozotocin and unilateral nephrectomy is an effective method for inducing experimental diabetic nephropathy in the ‘resistant’ C57Bl/6J mouse strain**

Melissa Uil^1^, Angelique M.L. Scantlebery^1^, Loes M. Butter^1^, Per W.B. Larsen^1^, Onno J. de Boer^1^, Jaklien C. Leemans^1^, Sandrine Florquin^1^, Joris J.T.H. Roelofs^1*^

^1^Department of Pathology, Academic Medical Center, University of Amsterdam, the Netherlands

^*^Corresponding author: j.j.roelofs@amc.nl

**Supplementary Figures**

**Supplementary Figure 1**


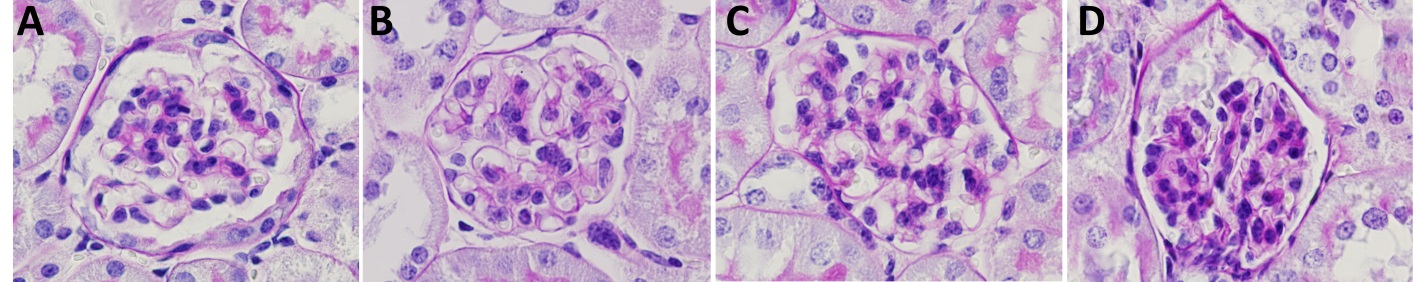


**S1. Mesangial matrix expansion**. Representative photographs are depicted for W mice (A), WS mice (B), WSU mice (C) and WU ice (D).

**Supplementary Figure 2**


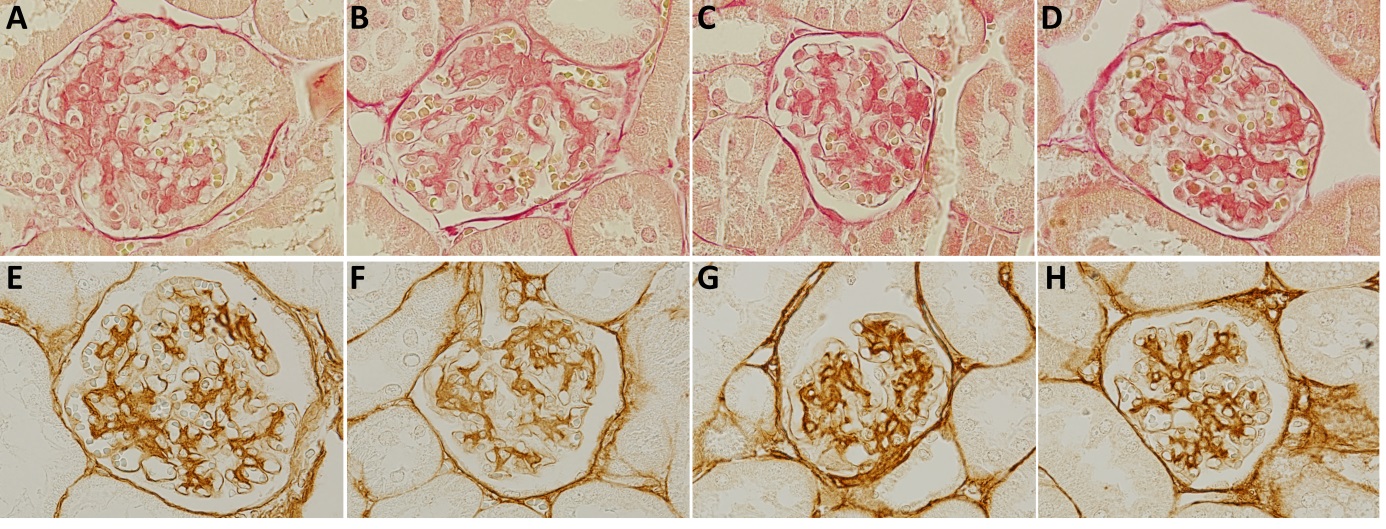


**S2.** **Glomerular collagen expression.** Representative photographs (40x magnification) of glomerular collagen deposition (from PSR staining) in W mice (**A**), WS mice (**B**), WSU mice (**C**) and WU mice (**D**). Representative photographs of glomerular collagen IV deposition in W mice (**E**), WS mice (**F**), WSU mice (**G**) and WU mice (**H**).

**Supplementary Figure 3**


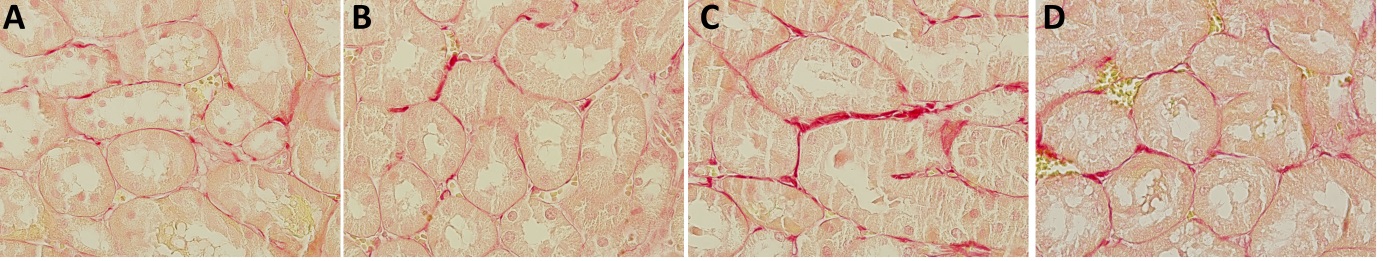


**S3. Cortical collagen deposition.** Representative photographs (40x magnification) of cortical collagen deposition (from PSR staining) in W mice (**A**), WS mice (**B**), WSU mice (**C**) and WU mice (**D**).
